# Results of a CIE Detector Response Intercomparison^1^

**DOI:** 10.6028/jres.095.041

**Published:** 1990

**Authors:** Douglas B. Thomas, Edward F. Zalewski

**Affiliations:** National Institute of Standards and Technology, Gaithersburg, MD 20899

**Keywords:** laboratory evaluation, laser, optical radiation, photodiode, spectral response

## Abstract

A total of fifteen laboratories participated in the CIE detector response intercomparison which was designed to assess the level of agreement among participating laboratories in the absolute measurement (with respect to SI) of photodetector response in the visible spectral region. Most participants were either commercial laboratories or university laboratories with the National Institute of Standards and Technology (NIST) serving as the host laboratory. Each laboratory determined the absolute response of each of two silicon photodiode radiometers which were designed for the intercomparison by NIST. Approximately two-thirds of the laboratories reported response values which agreed with the NIST values to within ±1.0% at the two wavelengths of 488 and 633 nm.^3^

## 1. Introduction

This report provides the final results of a detector response intercomparison under the aegis of CIE Technical Committee TC 2-06 on Absolute Spectral Responsivity of Detectors. Members of the Technical Committee are listed in Appendix B of this report.

The primary purpose of the intercomparison was to assess the level of agreement among participating laboratories in the absolute measurement (with respect to SI) of photodetector response (A/W) in the visible spectral region. The method chosen to accomplish this is to have these laboratories measure the absolute response of selected radiometersat two specific wavelengths near each end of the visible spectrum. The wavelengths selected are those of the helium-neon laser (632.8 nm) and the argon ion laser (488.0 nm).

The intercomparison was implemented on the basis of the National Institute of Standards and Technology (NIST) serving as the host laboratory and providing (a) the radiometers to be used in the intercomparison, (b) instructions to participating laboratories in the use of the radiometers in absolute response measurements, and (c) data analysis and a report of the results of the intercomparison.

The intercomparison was conducted in two stages: (1) intercomparison of U.S. laboratories and (2) intercomparison of laboratories outside of the United States. All participating laboratories except two are either commercial laboratories or university laboratories. The Electrotechnical Laboratory in Ibaraki, Japan and the Van Swinden Laboratory, The Netherlands, are national standards laboratories. The intercomparison was planned on the basis that NIST would measure the absolute response of all radiometers before shipment to the participating laboratories and then again after the radiometers were returned. The NIST absolute response value for each radiometer would be the average of the two NIST measurements.

## 2. The Radiometers

The radiometers used in this intercomparison were designed for ruggedness and ease of use and included commercially available silicon photodiodes. Each radiometer consists of a silicon photodiode and amplifier circuit mounted in a cylindrical aluminum housing and an external power supply.

Since it was expected that the majority of the laboratories would make their measurements using lasers, the photodiodes were not protected by a window. However, to protect each photodiode during non-use, the diodes were maintained in sealed compartments.

Two types of detectors were used: EG&G^4^ model UV-444B PN photodiodes and UDT model UV-100 inversion layer photodiodes. Radiometers PI-17, PI-19, PI-20, and PI-21 have the EG&G photodiodes while radiometers PI-25 through PI-32 have the UDT photodiodes. For the UDT photodiodes, a constant reverse bias voltage (4.5 V) was supplied by lithium batteries within each radiometer. The amplifier in each radiometer has gain settings from 10^4^ to 10^9^ V/A with accuracies of ±0.03% except for the 10^9^ range where it is ±0.5%.

Each participating U.S. laboratory received two radiometers: one with an EG&G photodiode and one with a UDT photodiode. The reason for requesting each laboratory to measure two radiometers is two-fold. (1) A second radiometer provides a backup for possible shipping damage and (2) there is a check on measurement repeatability.

After the first stage of the intercomparison was completed involving U.S. laboratories, a decision was made to use only the radiometers with the UDT photodiodes for the intercomparison involving laboratories in other countries. This decision was made when it was determined that the EG&G photodiodes exhibited a small but significant response drift at 488 nm over long periods of time (see sec. 4).

## 3. The Intercomparison

A total of six U.S. laboratories and nine laboratories in other countries participated in the intercomparison. Each laboratory was asked to complete a questionnaire concerning detailed information about their detector response measurement system and to use standard data forms for reporting their results. Tables 1 and 2 list the U.S. laboratories and the information each submitted about their measurements at 488 and 633 nm. Tables 3 and 4 list the corresponding information for laboratories in other countries. The information submitted covered eight measurement parameters: (1) absolute base (absolute standard(s) used), (2) standard deviation of the measurements, (3) number of measurements per radiometer, (4) type of radiation source used, (5) beam diameter of the source, (6) radiant power level at the radiometer, (7) ambient temperature during measurements, and (8) estimated uncertainty (with respect to SI) of the absolute standards used. Some laboratories used a single silicon photodiode as an absolute (standard) base for their measurements. The absolute response of these photodiodes was determined using the self-calibration method [1,2]. Two laboratories made measurements only at 633 nm. Of the fifteen laboratories participating in the intercomparison, five laboratories used lasers as radiation sources at both wavelengths and five used a tungsten lamp/filter/monochromator system at both wavelengths. The remaining laboratories used various combinations of these sources. Radiant power levels ranged from 0.16 *μ*W to 0.7 mW.

**Figure 1:**
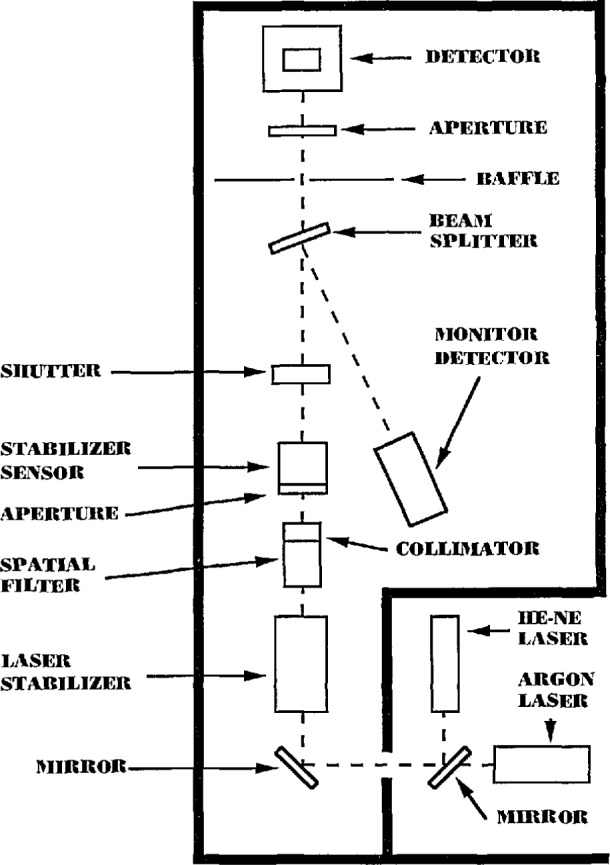
NIST laser based detector calibration facility.

The measurement system used at NIST for this intercomparison consists of He-Ne and Argon ion lasers, laser stabilizer, spatial filter, beam splitter, and a silicon photodiode monitor detector. Three UDT QED-200 absolute radiometers [3] were used as base standards. Figure 1 is an illustration of the system components. The NIST procedures for determining the absolute response of the intercomparison radiometers consisted basically of two steps: (1) measuring the ratio of the photocurrent of each UDT QED-200 radiometer to the photocurrent of the monitor detector at a particular laser power setting and (2) measuring the ratio of the photocurrent of the intercomparison radiometers to the monitor detector at the same power level in (1). Since the UDT QED-200 radiometers are 100% quantum efficient (with voltage bias) at the wavelengths and power levels stated, the power (watts) can be accurately measured and the absolute response (amperes/watt) of each of the intercomparison radiometers can be determined. Details concerning the system and the measurement procedure are further described in [4]. The NIST absolute base was compared to other international standards laboratories in a recent detector response intercomparison sponsored by the Consultative Committee on Photometry and Radiometry (CCPR) [5]. In the CCPR intercomparison, the absolute response of a select group of silicon photodiode radiometers were measured by 10 international standards laboratories and also by NIST which served as the host laboratory. The ratios of the NIST response values to the mean of the response values of the other participating laboratories were 1.0011±0.0035 and 1.0014±0.0037 at the two wavelengths of 488 and 633 nm, respectively.

Since the absolute response values reported by each of the participating laboratories were compared to the response values determined at NIST, it was essential for NIST to measure the response of each set of radiometers *before* it was shipped to the participating laboratory and then measured again *after* the radiometers were returned. The *before* and *after* measurements by NIST were made to determine if any significant changes occurred in the radiometers during shipment.

## 4. Data Analysis

Tables 5 and 6 list the laboratory designations, date of measurement, radiometer descriptions, and absolute responsivities reported by the U.S. laboratories and laboratories in other countries, respectively. Each set of response values for a participating laboratory includes the corresponding *before* and *after* values determined by NIST. The NIST value for each radiometer was taken as the average of the *before* and *after* respective values. The *before* and *after* NIST values indicate that some of the radiometers had undergone a small but significant change in response between shipments to and from the laboratories. For example, at 488 nm, the response value for radiometer PI-20 (laboratory C, table 5) decreased from 0.2814 to 0.2787 over the period 7/87 to 2/88 as measured by NIST. This is a decrease of 0.96%. All ratios reported represent an average of the *before* and *after* values.

Since three of the four radiometers with the EG&G type photodiode showed small but significant decreases in response at 488 nm over a 7-month period, it was decided to use only the radiometers with the UV-100 type photodiodes for the second phase of the intercomparison (foreign laboratories).

Table 7 is a listing of the participating laboratories by code letter, the absolute response values reported by each laboratory, the absolute response values as determined by NIST, and the ratios of the response values.

**Figure 2:**
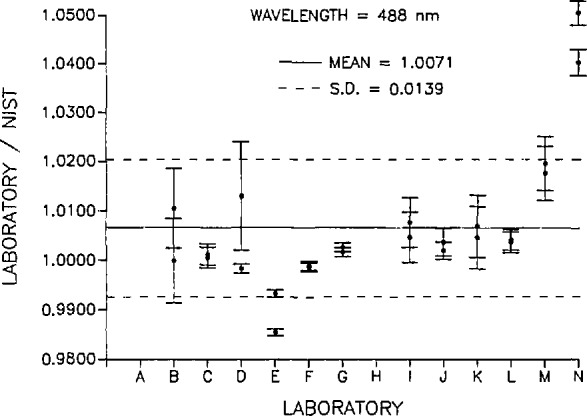
Ratio of the participant laboratory spectral response to that determined by NIST at 488 nm. The error bars indicate the quadrature summation of the measurement and absolute uncertainties of each participant laboratory, the *before/after* response change for each radiometer, and the NIST measurement and absolute uncertainties. The dashed lines indicate the standard deviation of the ratio values.

**Figure 3:**
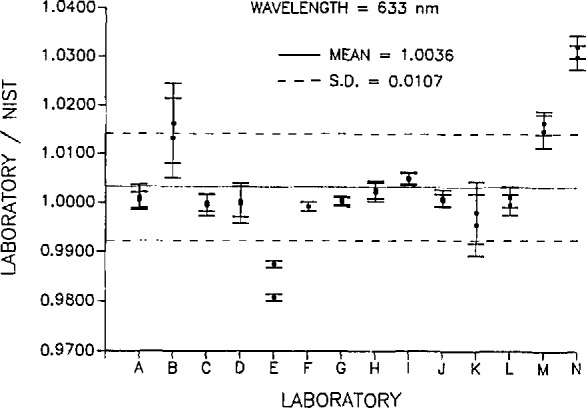
Ratio of the participant laboratory spectral response to that determined by NIST at 633 nm. The error bars indicate the quadrature summation of the measurement and absolute uncertainties of each participant laboratory, the *before/after* response change for each radiometer, and the NIST measurement and absolute uncertainties. The dashed lines indicate the standard deviation of the ratio values.

Figures 2 and 3 are plots of the ratios of the response values (A/W) determined by each of the participating laboratories to the respective response values (A/W) determined by NIST at 488 and 633 nm. The solid line on each plot is the mean of all the ratios at the respective wavelength and the dashed lines are the standard deviation of the mean. Table 8 is a summary of the standard deviations of the measurements and the estimated uncertainty (with respect to SI) of the absolute standards used by each of the participating laboratories. Also listed are the *before/after* change in absolute response for each detector as measured by NIST and the absolute response ratio uncertainty. The absolute response ratio uncertainty is the quadrature summation of the measurement and absolute uncertainties of each participant laboratory, the *before/after* response change for each radiometer, and the NIST measurement and absolute uncertainties. The error bars in figures 2 and 3 indicate the absolute response ratio uncertainty for each laboratory.

## 5. Conclusion

In general, it can be concluded that most of the response values reported by the laboratories were in good agreement with NIST. At 488 nm, the mean of all participating laboratories was 0.71% higher than the corresponding NIST values with a standard deviation of 1.39%. Similarly, at 633 nm, the mean of all laboratory values was higher than the NIST values by 0.36% with a standard deviation of 1.07%. All laboratories participating in this intercomparison (except laboratory N) reported values at both wavelengths within ±2.0% of the NIST values and nine of the 14 laboratories reported values at both wavelengths within ±1.0% of the NIST values. This can be considered good agreement among the laboratories when one considers the variety of sources, procedures, and testing environments involved in this intercomparison.

## Figures and Tables

**Table 1:** Participating U.S. laboratories. Wavelength=488 nm

	LLL	NIST	TEKX	UDT	UAZ	WEST
Absolute base	EGG	QED2	QED, UDT	QED2	QED2	
S.D. of measurements	0.24– 0.35%	0.01%	0.11%	0.039– 1.10%	0.003– 0.018%	
No. of meas./Radiometer	5	50	3	2	48	
Radiation source	TLF	ARL	TLM	BEN	TLF	
Beam diameter	OFA	4 mm	2 × 5	1 × 5	2 × 3	
Power level	3.7 × 10^−8^ W/cm^2^	0.5 mW	0.6 *µ*W	2.0 *µ*W	4.0 *µ*W	
Ambient temp. (°C)	22	25–26	23.0	21.0	21.0	
Est. abs. uncertainty	0.77%	0.10%	0.17%	0.07%	0.05%	

LLL—Lawrence Livermore National Laboratory, Livermore, California.

NIST—National Institute of Standards and Technology, Gaithersburg, Maryland (Host Laboratory).

TEKX—Tektronix Corporation, Beaverton, Oregon.

UDT—United Detector Technology, Hawthorne, California.

UAZ—University of Arizona, Tucson, Arizona.

WEST—Westinghouse Electric Corporation, Baltimore, Maryland.

EGG—EG&G UV-444-BQ Photodiode.

QED—UDT QED-100 Radiometer.

QED2—UDT QED-200 Radiometer.

UDT—UDT UV-100L Photodiode.

TLF—Tungsten lamp/Filter.

ARL—Argon ion laser.

HENE—Helium-neon laser.

TLM—Tungsten lamp/Monochromator.

BEN—Bentham M300 Monochromator.

1 × 5—1 × 5 mm rectangle.

2 × 3—2.5 × 3.5 mm rectangle.

2 × 5—2.5 × 5.0 mm rectangle.

OFA—Overfill of aperture.

**Table 2:** Participating U.S. laboratories. Wavelength = 633 nm

	LLL	NIST	TEKX	UDT	UAZ	WEST
Absolute base	EGG	QED2	QED, UDT	QED2	QED2	UDT, QED2 EGG
S.D. of measurements	0.28%	0.012%	0.015– 0.12%	0.3– 0.4%	0.018– 0.004%	0.26– 0.14%
No. of meas./Radiometer	5	50	6	2	64	3
Radiation source	TLF	HENE	TLM, HENE	BEN, HENE	TLF	HENE
Beam diameter	OFA	4 mm	2 × 5, 2.5 mm	1 × 5, 4 mm	2 × 3	2 mm
Power level	4.9 × 10^−8^ W/cm^2^	0.5 mW	0.6 *µ*W, 0.5 *µ*W	2.0 *µ*.W, 0.5 mW	11 *µ*W	0.44 mW
Ambient temp. (°C)	22	25–26	23.0	21.0	21.0	20.8
Est. abs. uncertainty	0.77%	0.10%	0.17%	0.07%	0.05%	0.05%

LLL—Lawrence Livermore National Laboratory, Livermore California.

NIST—National Institute of Standards and Technology, Gaithersburg, Maryland (Host Laboratory).

TEKX—Tektronix Corporation, Beaverton, Oregon.

UDT—United Detector Technology, Hawthorne, California.

UAZ—University of Arizona, Tucson, Arizona.

WEST—Westinghouse Electric Corporation, Baltimore, Maryland.

EGG—EG&G UV-444-BQ Photodiode.

QED—UDT QED-100 Radiometer.

QED2—UDT QED-200 Radiometer.

UDT—UDT UV-100L Photodiode.

TLF—Tungsten lamp/Filter.

ARL—Argon ion laser.

TLM—Tungsten lamp/Monochromator.

HENE—Helium-neon laser.

BEN—Bentham M300 Monochromator.

1 × 5—1 × 5 mm rectangle.

2 × 3—2.5 × 3.5 mm rectangle.

2 × 5—2.5 × 5.0 mm rectangle.

OFA—Overfill of aperture.

**Table 3:** Participating laboratories in other countries. Wavelength=488 nm

	CIP	ETL	HAM	LCIE	LNE	MAT	KROC	UDI	VSL
Absolute base	PSP	HAM2		ASP	TSP	HAM2	HAM3	EGG	QED2
S.D. of measurements	0.17– 0.21%	0.04%		0.6%	0.11– 0.18%	0.02%	0.52%	0.12– 0.04%	0.007– 0.011%
No. of meas./Radiometer	10	4		6	27,33	10	5	8–12	75
Radiation source	ARL	TLM		TLM	TLM	ARL	TLM	ARL	ARL
Beam diameter	4 mm	2 × 3		5 mm	6 mm	3 mm	7 mm	0.6 mm	4 mm
Power level	0.02 mW	0.16 *µ*W		20 *µ*W	1.5 *µ*W	0.19 mW	2.4 × 10^−3^ W/m^2^	0.3 mW	0.7 mW
Ambient temp. (°C)	18	23		23	23	25	23	21	23
Est. abs. uncertainty	0.17%	0.07%		0.20%	0.11 0.22%	0.07%	0.17%	0.50%	0.20%

CIP—Central Institute of Physics, Magurele-Bucharest, Romania.

ETL—Electrotechnical Laboratory, Ibaraki, Japan.

HAM—Hamamatsu Photonics K.K., Hamamatsu City, Japan.

LCIE—L.C.I.E., Fontenay-aux-Roses, France.

LNE—Laboratoire National D’Essais, Paris, France.

MAT—Matsushita Electric Industrial Co. Ltd., Moriguchi Osaka, Japan.

KROC—PR C Krochmann GMBH, Berlin, West Germany.

UDI—University College, Dublin, Ireland.

VSL—Van Swinden Laboratory, Delft, The Netherlands.

PSP—*pn* Silicon photodiode (Romanian).

HAM2—Hamamatsu S 1337 Photodiode.

HAM3—Hamamatsu S 1227 Photodiode.

ASP—Silicon photodiode.

TSP—Three silicon photodiodes.

EGG—EG&G UV-444B Photodiode.

QED2—UDT QED-200 Radiometer.

ARL—Argon ion laser.

TLM—Tungsten lamp/Monochromator.

2 × 3—2 × 3 mm rectangle.

**Table 4:** Participating laboratories in other countries. Wavelength = 63 3 nm

	CIP	ETL	HAM	LCIE	LNE	MAT	KROC	UDI	VSL
Absolute base	PSP	HAM1	HAM2	ASP	TSP	HAM2	HAM3	EGG	QED2
S.D. of measurements	0.17–0.14%	0.02%	0.07–0.04%	0.6%	0.10–0.08%	0.02%	0.3–0.15%	0.07–0.08%	0.02–
No. of meas./Radiometer	10	3	10	6	21,28	10	5	6	75
Radiation source	HENE	HENE	HENE	TLM	TLM	HENE	TLM	HENE	HENE
Beam diameter	4 mm	1 mm	1.5 mm	5 mm	6 mm	3 mm	7 mm	0.6 mm	4 mm
Power level	0.1 mW	40 *µ*W	25 *µ*W	30*µ*W	2 *µ*W	0.3 mW	1.1 × 10^−2^ W/m^2^	0.6 mW	0.7 mW
Ambient temp. (°C)	18	23	25	23	23	25	23	18	23
Est. abs. uncertainty	0.17%	0.07%	0.17%	0.20%	0.12 0.09%	0.07%	0.17%	0.08%	0.20%

CIP—Central Institute of Physics, Magurele-Bucharest, Romania.

ETL—Electrotechnical Laboratory, Ibaraki, Japan.

HAM—Hamamatsu Photonics K.K., Hamamatsu City, Japan.

LCIE—L.C.I.E., Fontenay-aux-Roses, France.

LNE—Laboratoire National D’Essais, Paris, France.

MAT—Matsushita Electric Industrial Co. Ltd., Moriguchi Osaka, Japan.

KROC—PRC Krochmann GMBH, Berlin, West Germany.

UDI—University College, Dublin, Ireland.

VSL—Van Swinden Laboratory, Delft, The Netherlands.

PSP—*pn* Silicon photodiode (Romanian).

HAM1—Hamamatsu S 1723 Photodiode.

HAM2—Hamamatsu S 1337 Photodiode.

ASP—Silicon photodiode.

TSP—Three silicon photodiodes.

HAM3—Hamamatsu S 1227 Photodiode.

EGG—EG&G UV-444B Photodiode.

QED2—UDT QED-200 Radiometer.

HENE—Helium-neon laser.

TLM—Tungsten lamp/Monochromator.

**Table 5:** U.S. laboratories

Laboratory	Date	Responsivity (488 nm)		Responsivity (633 nm)	
				**PI-20**	**PI-25**
NIST	7/87			0.4551	0.4152
LAB A	7/87			0.4555	0.4154
NIST	7/87			0.4548	0.4153
		**PI-21**	**PI-27**	**PI-21**	**PI-27**
NIST	7/87	0.2830	0.2994	0.4550	0.4140
LAB B	9/87	0.2857	0.2990	0.4608	0.4208
NIST	12/87	0.2824	0.2986	0.4546	0.4141
		**PI-20**	**PI-25**	**PI-20**	**PI-25**
NIST	7/87	0.2814	0.2982	0.4548	0.4153
LABC	9/87	0.2802	0.2987	0.4547	0.4151
NIST	2/88	0.2787	0.2984	0.4545	0.4153
		**PI-19**	**PI-28**	**PI-19**	**PI-28**
NIST	7/87	0.2596	0.2965	0.4472	0.4165
LAB D	11/87	0.2570	0.3006	0.4468	0.4168
NIST	2/88	0.2551	0.2969	0.4467	0.4169
		**PI-17**	**PI-30**	**PI-17**	**PI-30**
NIST	7/87	0.2849	0.3021	0.4580	0.4108
LAB E	12/87	0.2799	0.3003	0.4521	0.4027
NIST	2/88	0.2830	0.3025	0.4576	0.4105

**Table 6:** Laboratories in other countries

Laboratory	Date	Responsivity (488 nm)		Responsivity (633 nm)	
		**PI-28**	**PI-31**	**PI-28**	**PI-31**
NIST	2/88	0.2969	0.3014		
NIST	8/88			0.4169	0.4125
LAB F	10/88	0.2964	0.3008	0.4166	0.4124
NIST	12/88			0.4169	0.4129
NIST	1/89	0.2967	0.3009		
		**PI-28**	**PI-31**	**PI-28**	**PI-31**
NIST	2/88	0.2969	0.3014		
NIST	8/88			0.4169	0.4125
LAB G	11/88	0.2973	0.3019	0.4170	0.4129
NIST	12/88			0.4169	0.4129
NIST	1/89	0.2967	0.3009		
				**PI-28**	**PI-31**
NIST	8/88			0.4169	0.4125
LAB H	12/88			0.4178	0.4138
NIST	12/88			0.4169	0.4129
		**PI-25**	**PI-32**	**PI-25**	**PI-32**
NIST	2/88	0.2983	0.2599		
NIST	8/88			0.4153	0.4515
LAB I	11/88	0.2999	0.2622	0.4171	0.4538
NIST	12/88			0.4148	0.4514
NIST	1/89	0.2987	0.2605		
		**PI-28**	**PI-31**	**PI-28**	**PI-31**
NIST	12/88			0.4169	0.4129
NIST	1/89	0.2967	0.3009		
LAB J	5/89	0.2972	0.3020	0.4172	0.4130
NIST	8/89			0.4168	0.4127
NIST	9/89	0.2966	0.3009		
		**PI-28**	**PI-31**	**PI-28**	**PI-31**
NIST	12/88			0.4169	0.4129
NIST	1/89	0.2967	0.3009		
LAB K	7/89	0.2980	0.3030	0.4160	0.4110
NIST	8/89			0.4168	0.4127
NIST	9/89	0.2966	0.3009		
		**PI-25**	**PI-32**	**PI-25**	**PI-32**
NIST	12/88			0.4148	0.4514
NIST	1/89	0.2987	0.2605		
LAB L	6/89	0.2995	0.2617	0.4153	0.4511
NIST	8/89			0.4148	0.4512
NIST	9/89	0.2982	0.2607		
		**PI-27**	**PI-29**	**PI-27**	**PI-29**
NIST	8/88			0.4145	0.4149
NIST	2/89	0.2989	0.2991		
LAB M	4/89	0.3043	0.3049	0.4205	0.4216
NIST	8/89			0.4144	0.4147
NIST	9/89	0.2992	0.2988		
		**PI-26**	**PI-30**	**PI-26**	**PI-30**
NIST	7/88	0.2667	0.3020		
NIST	8/88			0.4454	0.4108
LAB N	12/88	0.2805	0.3144	0.4583	0.4240
NIST	8/89			0.4446	0.4107
NIST	9/89	0.2672	0.3023		

**Table 7:** Response ratios

Laboratory(RAD. #)	488 nm			633 nm		
	Resp. Lab.	Resp. NIST	Lab./NIST	Resp. Lab.	Resp. NIST	Lab./NIST
A (PI-20)				0.4555	0.4550	1.0011
A (PI-25)				0.4154	0.4152	1.0005
B (PI-21)	0.2857	0.2827	1.0106	0.4608	0.4548	1.0132
B (PI-27)	0.2990	0.2990	1.0000	0.4208	0.4141	1.0162
C (PI-20)	0.2802	0.2806	0.9986	0.4547	0.4547	1.0000
C (PI-25)	0.2987	0.2983	1.0013	0.4151	0.4153	0.9995
D (PI-19)	0.2570	0.2570	1.0000	0.4468	0.4469	0.9998
D (PI-28)	0.3006	0.2967	1.0131	0.4168	0.4167	1.0002
E (PI-17)	0.2799	0.2836	0.9870	0.4521	0.4578	0.9875
E (PI-30)	0.3003	0.3023	0.9934	0.4027	0.4106	0.9808
F (PI-28)	0.2964	0.2968	0.9987	0.4166	0.4169	0.9993
F (PI-31)	0.3008	0.3011	0.9990	0.4124	0.4127	0.9993
G (PI-28)	0.2973	0.2968	1.0017	0.4170	0.4169	1.0002
G(PI-31)	0.3019	0.3011	1.0027	0.4129	0.4127	1.0005
H (PI-28)				0.4178	0.4169	1.0022
H (PI-31)				0.4138	0.4127	1.0027
I (PI-25)	0.2999	0.2985	1.0047	0.4171	0.4151	1.0048
I (PI-32)	0.2622	0.2602	1.0077	0.4538	0.4515	1.0051
J (PI-28)	0.2972	0.2966	1.0020	0.4172	0.4168	1.0010
J (PI-31)	0.3020	0.3009	1.0037	0.4130	0.4128	1.0005
K (PI-28)	0.2980	0.2966	1.0047	0.4160	0.4168	0.9981
K (PI-31)	0.3030	0.3009	1.0070	0.4110	0.4128	0.9956
L (PI-25)	0.2995	0.2984	1.0037	0.4153	0.4148	1.0012
L (PI-32)	0.2617	0.2606	1.0042	0.4511	0.4513	0.9996
M (PI-27)	0.3043	0.2990	1.0177	0.4205	0.4144	1.0147
M (PI-29)	0.3049	0.2990	1.0197	0.4216	0.4148	1.0164
N (PI-26)	0.2805	0.2670	1.0506	0.4583	0.4450	1.0299
N (PI-30)	0.3144	0.3022	1.0404	0.4240	0.4108	1.0321

**Table 8:** Summary of uncertainties

Laboratory	Measurement standard deviation (1 sigma)	Absolute uncertainty (1 sigma)	Before/After response change (%/100)	Ratio uncertainty (1 sigma)
488 nm				

LLL	0.0024	0.0077	0.0021	0.0084
	0.0035	0.0077	0.0027	0.0089
N1ST	0.0001	0.0010		
	0.0001	0.0010		
TEKX	0.0011	0.0017	0.0096	0.0099
	0.0011	0.0017	0.0007	0.0024
UDT	0.00039	0.0007	0.0175	0.0175
	0.0110	0.0007	0.0013	0.0111
UAZ	0.00003	0.0005	0.0067	0.0068
	0.00018	0.0005	0.0013	0.0017
CIP	0.0017	0.0017	0.0019	0.0032
	0.0021	0.0017	0.0010	0.0031
ETL	0.0004	0.0007	0.0007	0.0015
	0.0004	0.0007	0.0017	0.0021
LCIE	0.0060	0.0020	0.0003	0.0064
	0.0060	0.0020	0.0000	0.0064
LNE	0.0011	0.0011	0.0003	0.0019
	0.0018	0.0022	0.0000	0.0030
MAT	0.0002	0.0007	0.0007	0.0014
	0.0002	0.0007	0.0017	0.0021
KROC	0.0052	0.0017	0.0010	0.0057
	0.0052	0.0017	0.0010	0.0057
UDI	0.0012	0.0050	0.0013	0.0054
	0.0004	0.0050	0.0023	0.0056
VSL	0.00007	0.0020	0.0017	0.0028
	0.00011	0.0020	0.0008	0.0024

633 nm				

LLL	0.0028	0.0077	0.0009	0.0083
	0.0028	0.0077	0.0002	0.0083
NIST	0.00012	0.0010		
	0.00012	0.0010		
TEKX	0.00015	0.0017	0.0007	0.0021
	0.0012	0.0017	0.0000	0.0023
UDT	0.0040	0.0007	0.0011	0.0043
	0.0030	0.0007	0.0010	0.0034
UAZ	0.00018	0.0005	0.0009	0.0014
	0.00004	0.0005	0.0007	0.0013
WEST	0.0026	0.0005	0.0007	0.0029
	0.0014	0.0005	0.0002	0.0018
CIP	0.0017	0.0017	0.0018	0.0032
	0.0014	0.0017	0.0002	0.0024
ETL	0.0002	0.0007	0.0000	0.0012
	0.0002	0.0007	0.0010	0.0016
HAM	0.0007	0.0017	0.0000	0.0021
	0.0004	0.0017	0.0010	0.0022
LCIE	0.0060	0.0020	0.0002	0.0064
	0.0060	0.0020	0.0005	0.0064
LNE	0.0010	0.0012	0.0002	0.0019
	0.0008	0.0009	0.0005	0.0016
MAT	0.0002	0.0007	0.0000	0.0012
	0.0002	0.0007	0.0010	0.0016
KROC	0.0030	0.0017	0.0002	0.0036
	0.0015	0.0017	0.0005	0.0025
UDI	0.0007	0.0008	0.0012	0.0019
	0.0008	0.0008	0.0002	0.0015
VSL	0.0002	0.0020	0.0000	0.0022
	0.0002	0.0020	0.0005	0.0023

## Footnotes

4 Certain commerical equipment, instruments, or materials are identified in this paper to specify adequately the experimental procedure. Such identification does not imply recommendation or endorsement by the National Institute of Standards and Technology or by the CIE, nor does it imply that the materials or equipment identified are necessarily the best available for the purpose.

## 6. Appendix A

### Resumé

Quinze laboratories situes tant aux Etats-Unis que dans d’autres pays du monde entire ont pris part, dans le cadre de la CIE, a une comparaison de mesures de sensibilite de detecteurs qui avait pour but de determiner le niveau d’accord existant entre les laboratoires participants, pour la mesure de la sensibilite absolue (par rapport au SI) des photodetecteurs dans le domaine visible. La plupart des participants etaient des laboratories industriels ou des laboratories universitaires. Le National Institute of Standards and Technology (NIST) jouait le role de laboratoire pilote. Chaque laboratoire a determine la sensibilite absolute de deux radiometres equipes de photodiodes au silicium, et specialement realises pour cette comparaison par le NIST. Les resultats fournis par environ les 2/3 des laboratoires sont en accord avec ceux du NIST dans la limite d’incertitude de ±1% pour les longueurs d’onde de 488 et 633 nm.

### Zusammenfassung

Insqesamt fuenfzehn Laboratorien haben an CIE Vergleichsmessungen von optischen Strahlungsempfaengern teilgenommen. Der Vergleich bezweckte die Uebereinstimmung unter den teilnehmenden Laboratorien in der Absolutmessung (relativ zu SI Einheiten) der Empfindlichkeit von Halbleiter-Empfaengern im sichtbaren Spektralgebiet zu bestimmen. Die Mehrzahl der Teilnehmer waren Industrie- oder Universitaetslaboratorien. Das U.S. National Institute of Standards and Technology (NIST) war das Zentrallaboratorium. Jedes Labor bestimmte die absolute Empfindlichkeit von je zwei speziell fuer den Vargleich entwickelten NIST Radiometern mit Silizium-Photodioden. Ungefaehr zwei Drittel der von den einzelnen Laboratorien gemessenen Empflichkeiten fielen innerhalb ± 1% der NIST Werte bei 488 und 633 nm.

## 7. Appendix B

The following is a listing of the members of CIE Technical Committee TC 2-06 on Absolute Spectral Responsivity of Detectors. An asterisk (*) identifies those members who made the detector measurements for this intercomparison.

* Philip Armatis, Lawrence Livermore National Laboratory, Livermore, California, United States.
* Douglas Thomas, National Institute of Standards and Technology, Gaithersburg, Maryland, United States.

Albert Parr, National Institute of Standards and Technology, Gaithersburg, Maryland, United States.

Edward Zalewski, formerly of the National Institute of Standards and Technology, Gaithersburg, Maryland, United States.

* Ken Futornick, Tektronix Corporation, Beaverton, Oregon, United States.
* Richard Duda, United Detector Technology, Hawthorne, California, United States.
* James Palmer, University of Arizona, Tucson, Arizona, United States.
* Carroll Hughes III, Westinghouse Electric Corporation, Baltimore, Maryland United States.

James Christy, Hughes Electronics Corp., Tucson, Arizona, United States.

Ted Schrode, United States.

Kurt Scott, Atlas Electric Devices Company, Chicago, Illinois, United States.

* Dan Sporea, Central Institute of Physics, Magurele-Bucharest, Romania.
* Yasuo Mishima, Electrotechnical Laboratory, Ibaraki, Japan.
* Keiji Suyama, Hamamatsu Photonics K.K., Hamamatsu City, Japan.
* Yoshihiro Ohno, Matsushita Electric Industrial Co. Ltd., Moriguchi Osaka, Japan.
* B. Jean, Laboratoire Central des Industries Electriques, Fontenay-aux-Roses, France.
* Beatrice Chommeloux, Laboratoire National D’Essais, Paris, France.

Brigitte Mercier, Institute National de Metrologie, Paris, France.

*Gyorgy Czibula, PRC Krochmann GMBH, Berlin, West Germany.

J. Krochmann, PRC Krochmann GMBH, Berlin, West Germany.

*Eon O’Mongain, University College, Dublin, Ireland.

Maurice Goodman, University College, Dublin, Ireland.

*Jan de Vreede, Van Swinden Laboratory, Delft, The Netherlands.

Pieter Bloembergen, Van Swinden Laboratory, Delft, The Netherlands.

Antonio Corrons, Institute de Optica, Madrid, Spain.

Dominique Crommelynck, Royal Meteorological Institute of Belgium, Brussels, Belgium.

John Moore, National Physical Laboratory, Teddington, United Kingdom.

Juraji Zatkovic, Czechoslovak Institute of Metrology/CSMO, Bratislava, Czechoslovakia.

F. Hengstberger, NPRL/SCIR, Pretoria, South Africa.

Li Tong-Bao, National Institute of Measurement and Testing Technology, Chengdu, China.
